# A new strategy for directly calculating the minimum eigenvector of matrices without diagonalization

**DOI:** 10.1038/s41598-020-60103-5

**Published:** 2020-02-25

**Authors:** Wei Pan, Jing Wang, Deyan Sun

**Affiliations:** Department of Physics, East China Normal University, 200241 Shanghai, China

**Keywords:** Physics, Condensed-matter physics, Quantum physics

## Abstract

The diagonalization of matrices may be the top priority in the application of modern physics. In this paper, we numerically demonstrate that, for real symmetric random matrices with non-positive off-diagonal elements, a universal scaling relationship between the eigenvector and matrix elements exists. Namely, each element of the eigenvector of ground states linearly correlates with the sum of matrix elements in the corresponding row. Although the conclusion is obtained based on random matrices, the linear relationship still keeps for non-random matrices, in which off-diagonal elements are non-positive. The relationship implies a straightforward method to directly calculate the eigenvector of ground states for one kind of matrices. The tests on both Hubbard and Ising models show that, this new method works excellently.

## Introduction

Without any doubt, a lot of scientific problems are directly related to matrix algebra. Obtaining eigenvalues or eigenvectors of matrices is one of the basic tasks in many fields of science and technology. The significance of matrices is especially prominent in modern physics. Almost all quantum problems come down to the diagonalization of matrices in principle. A Hamiltonian matrix contains all the information of a corresponding quantum system, and the density matrix reflects all thermal properties of a system at finite temperatures. However the diagonalization of an arbitrary matrix for many physical systems of practical interests is definitely not an easy stuff. Especially in condensed matter and statistical physics, the number of particles usually has the magnitude of the Avogadro constant, correspondingly the many-body Hamiltonian matrix may quickly become a hopeless scale, which is too large to be diagonalized by any conventional mathematical method.

Over half a century, great efforts have been carried on the matrix diagonalization, and remarkable progresses have been made associated with the rapid development of modern computational technology. Speaking limited to physics, many diagonalization methods have been proposed, such as, exact diagonalization method^1–5^, quantum Monte-Carlo^6–12^, and the density matrix renormalization group^13–17^, *etc*. Even so, for almost all real physics systems, the diagonalization of matrices is still an impossible mission even with the help of modern computers.

When the direct diagonalization becomes an impractical task, one may naturally ask whether other feasible methods exist? Obviously, for an irreducible real symmetric matrix, once all of the matrix elements have been defined, the eigenvalues and eigenvectors are mathematically determined in principle. And any reducible matrix can always be simplified into several irreducible matrices. Thus the matrix elements essentially contain complete informations about the eigenvalues and eigenvectors. One possible idea is to establish an immediate connection between eigenvectors and matrix elements. If this kind of connection can be figured out, it may be an appealing method for matrix diagonalization. Certainly, it is not easy to obtain the possible connection, because few precedents can be followed to realize this idea. Fortunately, the strategy adopted in Big Data analysis and Machine Learning is heuristic, which can be used for reference^18^. In Big Data analysis and Machine Learning^19,20^, one usually makes predictions or decisions based on vast data sets without being programmed to perform the task. For example, in recent studies on many-body quantum systems, the physical properties are predicted without explicitly diagonalizing Hamiltonian matrices^18–25^.

Enlightened by many successful cases in the machine learning or Big Data analysis, we expect that, the connection between matrix elements and eigenvectors could be pried through the deep analysis for an enormous number of matrices. Here as a first attempt, we have focused on the random matrices (RMs), which were introduced in 1955 by Wigner^26^. RMs are common and important in many fields of physics. In quantum chaos, the Bohigas-Giannoni-Schmit conjecture is closely related to RMs^27^. In quantum optics, transformations described by RMs are crucial for demonstrating the advantage of quantum over classical computation (see, e.g., in refs. ^28,29^). In condensed matter physics, the fractional quantum Hall effect^30^, Anderson localization^31^, quantum dots^32^, superconductors^33^ and spin glasses^34,35^ are connected to RMs too. More multi-applications of RMs in physics can be found, for instance, in refs. ^36–41^.

In this work, we have systematically studied random real symmetric matrices with non-positive off-diagonal elements (hereafter labeled as RRSMs). It should be pointed out that, a matrix element can either be negative or zero. When the number of zero elements in a matrix is big, it is usually called as a sparse matrix, otherwise it is a dense matrix. In this work, we have studied both dense matrices and sparse matrices. The details regarding to produce a RRSM are described in the section of Methods. If the value of a matrix element is interpreted as the scattering amplitude as what does in quantum physics, RRSMs describe a system having the random scattering amplitudes. The choice of random matrices is also in order to make our conclusion having the universality, since random matrices cover all the possibilities in principle. At least for this kind of matrices, we have found a strong correlation between the eigenvector of the minimum eigenvalue (EME) and the sum of matrix elements (SME) in corresponding row. This result implies a new method for diagonalizations of RRSMs regardless of matrix dimension. The achievements of this work can also shed light on the solution of other complex matrices.

## Results

### Notations

A RRSM is denoted by *H*, and its dimension by *N*. *H*_*ij*_ represents the matrix element in the *i*-th row and the *j*-th column. For the sake of convenience, we assume that *H* is a Hamiltonian matrix of a certain quantum system. This assumption is just for convenience, does not alter our conclusion. Assuming orthogonal complete basis being |*e*_*i*_ > , where the subscript *i* refers the *i*-th basis, the projection of |*e*_*i*_ > on EME (|*G* > ) reads as *g*_*i*_, i.e., *g*_*i*_ = < *G*|*e*_*i*_ > and |*G* > = ∑_*i*_*g*_*i*_|*e*_*i*_ > .

Physically, the matrix element *H*_*ij*_ represents the scattering strength between the states |*e*_*i*_ > and |*e*_*j*_ > . When *H*_*ij*_ < 0, this scattering tends to reduce the total energy. Therefore, the sum of all scatters, namely *S*_*i*_ = ∑_*j*_*H*_*ij*_, should account for the major contribution of the state |*e*_*i*_ > to the ground state. Since our goal is to obtain the ground state, *i*. *e*. , to reduce the energy as low as possible, it is better to give a larger weight to the state which is corresponding to a smaller *S*_*i*_. Thus, we intuitively conjecture that the coefficients (*g*_*i*_) are correlated to *S*_*i*_, and the smaller (*i*. *e*. , the more negative) value of *S*_*i*_ corresponds to the larger value of *g*_*i*_. In the following context, we will demonstrate this conjecture and find the generic relationship. For better comparison among all matrices, *g*_*i*_ is re-scaled according to the normalization condition, and *S*_*i*_ is also normalized based on ${\sum }_{i}{S}_{i}^{2}=1$.

### Random matrices

**Figure 1:**
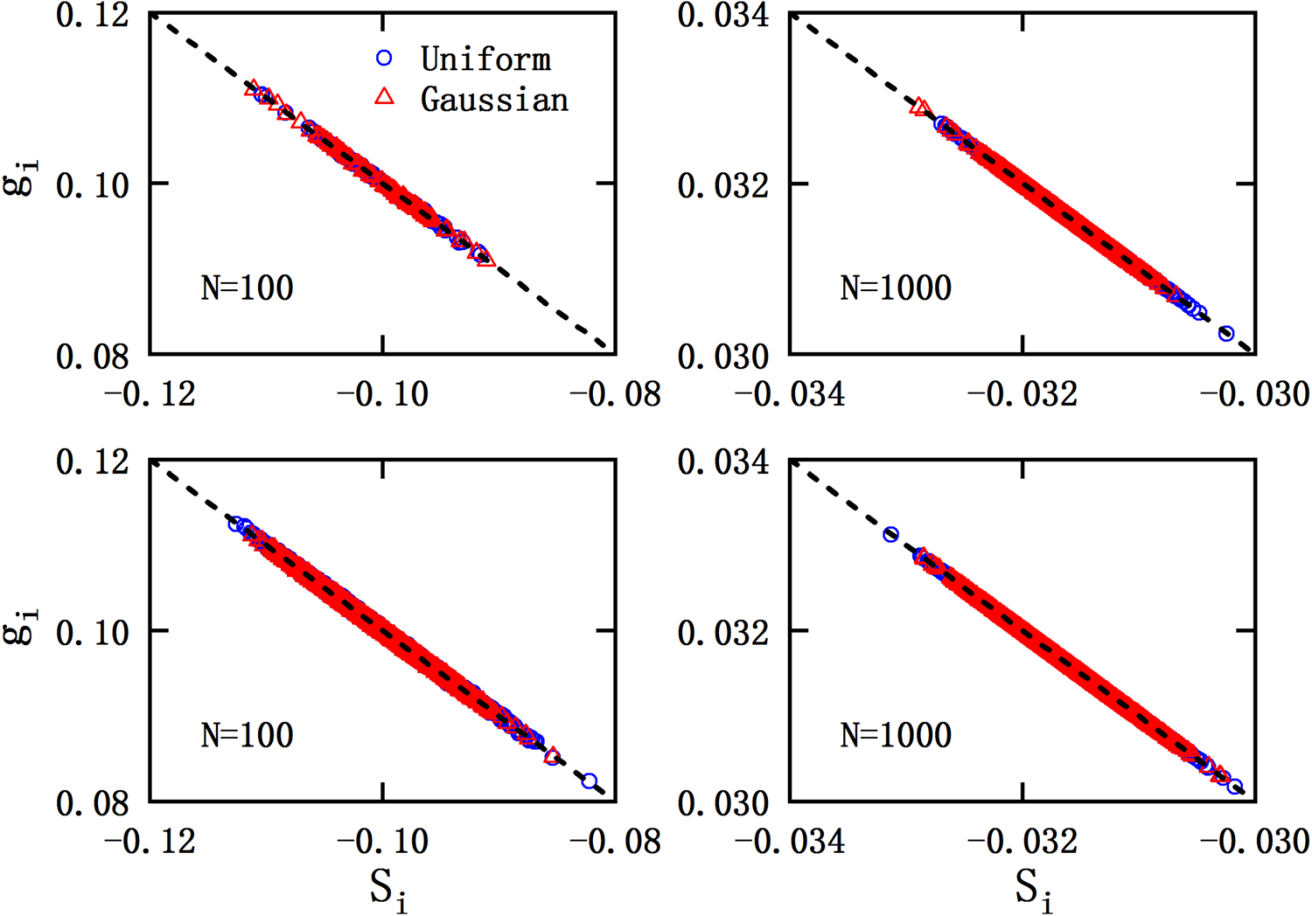
Elements of eigenvector for the minimum eigenvalue (*g*_*i*_) versus the sum of matrix elements in corresponding row (*S*_*i*_). Upper panels: one arbitrarily chosen matrix with uniform (blue ◦) and Gaussian (red △)distributions. Lower panels: all studied matrices with uniform (blue ◦) and Gaussian (red △) distributions. For all cases, an evident linear relationship can be observed.

 Figure. 1 shows the element of EME (*g*_*i*_) versus SME in corresponding row (*S*_*i*_) for both uniform (blue ◦) and Gaussian (red △) distributions, where all matrices are dense (*ρ* ~ 1). In the upper panels of this figure, the results for an arbitrarily chosen RRSM with the dimension of 100 (left) and 1000 (right) are presented. It can be seen that, *g*_*i*_ decreases with the increase of *S*_*i*_. The correlation between *g*_*i*_ and *S*_*i*_ shows an almost perfect linearity. The solid lines are the best fittings to the data, which produce a slope of −1 and intercept of 0. This correlation does not merely appear in several individual matrices, but in all the studied matrices, as shown in the lower panels of Fig. 1. It needs to be pointed out that, although Fig. 1 only presents the results of RRSMs with two specific dimensions (N = 100 and 1000), all the studied RRSMs exhibit the same linear scaling.

**Figure 2:**
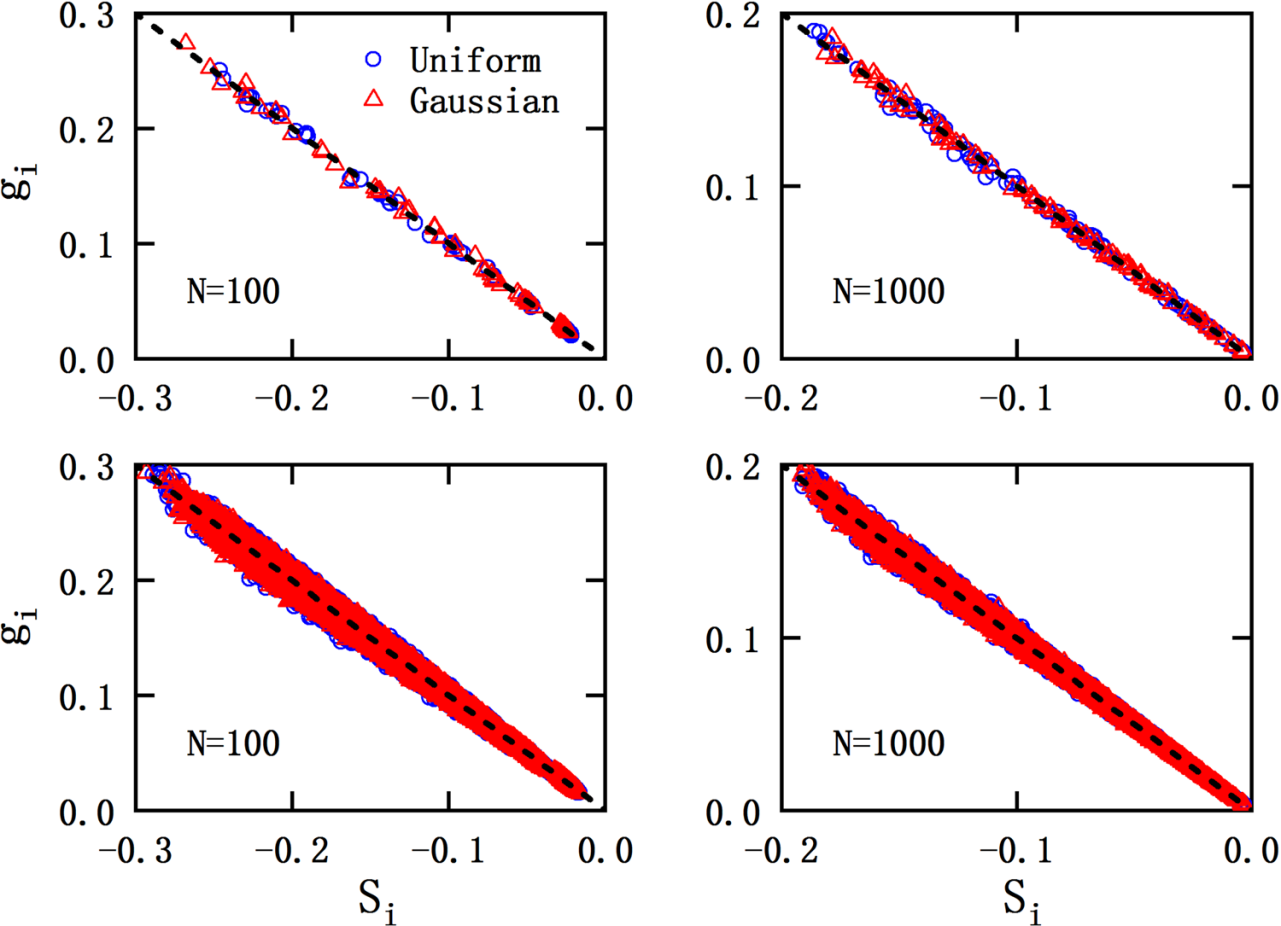
Similar to Fig. 1, but the amplitudes of matrix elements in some rows are enlarged or reduced. In this case, the linear relationship is still kept.

It is easy to demonstrate that, this linear relationship will not break down if the magnitudes of all matrix elements change simultaneously. The next question is, what would happen if the magnitude of matrix elements in some rows is significantly larger or smaller than those in other rows? Our results show that the linear relationship still keeps. In Fig. 2, the results are extended to these cases, in which matrix elements in several rows are much larger or smaller than others. The points corresponding to the enlarged (reduced) rows locate in the top (bottom) area in Fig. 2. One can see that, the correlation still remains linearity with a slope of −1 and intercept of 0. And the linear behavior does not break down no matter how many rows being enlarged or reduced.

From Figs. 1 and 2, we notice a remarkable feature, namely the linear relationship does not show evident deviation when the dimension of matrices increases. This point is extremely important for practical applications, because we are not only interested in the linear relationship, but also concern the possible applications of this relationship for large scale matrices. Obviously we can not extend our calculations to all high dimensional matrices. However, if we know how the deviation from linearity changes with the increase of matrix dimension, we are able to predict the validity at higher dimensions. To check this point, the root-mean-squared (*rms*) deviation from linearity is calculated, which reads:

1 $$rms=\sqrt{\frac{{\sum }_{i}^{N}{({g}_{i}-(-{S}_{i}))}^{2}}{N}}$$

where *N* is the dimension of matrix.

**Figure 3:**
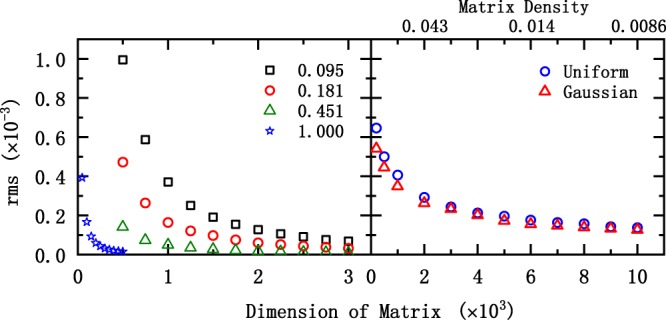
The root-mean-squared deviation from linearity (*rms*) as the function of matrix dimension. Left panel: for matrices with fixed density (*ρ*) of 1.000 (blue ☆), 0.095 (black □), 0.181 (red ◦) and 0.451 (green △). Right panel: matrices with density (*ρ*) varying as $\sim \frac{1}{N}$, where *N* is the matrix dimension. For all cases, the deviation decreases dramatically as the increase of matrix dimension, and quickly stabilizes at a small value.

In the left panel of Fig. 3 we have depicted *rms* deviations from linearity as function of the matrix dimension both for dense and sparse matrices, where the density of matrices is chosen as 1.000 (blue ☆), 0.095 (black □), 0.181 (red ◦) and 0.451 (green △). It is encouraging that, both dense and sparse matrices exhibit the similar trends, namely *rms* deviation decreases dramatically as the dimension of matrices increasing, and quickly stabilizes at a very small value. At the same dimension (*N*), *rms* deviation from linearity for sparse matrices is a little bit larger than that for dense ones.

In many physical systems of practical interests, the density of matrix decays with its dimension as $\rho  \sim \frac{1}{N}$. The right panel of Fig. 3 shows the results for this kind of matrices. It is more encouraging that the deviation is still convergent at large matrix dimensions in this case. Although we can not examine all the matrices with non-positive elements, the matrices considered in this work are quite general and sufficiently large in amounts, we believe that the linear relationship should be universal for this kind of matrices. We can conjecture in advance that the linear relationship may become strict as the matrix dimension approaching to infinite, if the matrix density is fixed.

**Figure 4:**
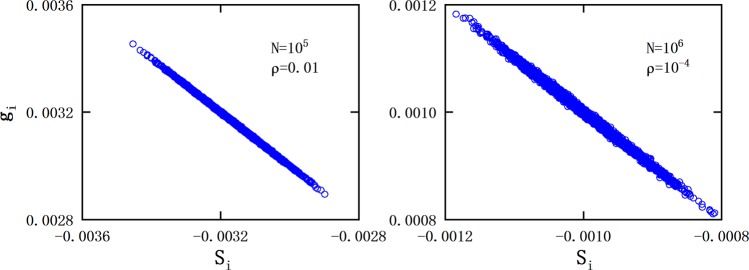
Similar to Fig. 1, but the matrix dimension is equal to 10^5^ (left panel) and 10^6^ (right panel), with the matrix density being *ρ* = 0.01 and 10^−4^ respectively.

In order to further check the influence of matrix dimensions on scaling relationships, we have calculated two larger random matrices (N = 10^5^ with *ρ* = 0.01, and N = 10^6^ with *ρ* = 10^−4^). Figure 4 presents the results for the two larger scale matrices. It is clear that, the scaling relationship does hold well for the two larger scale matrices. More importantly, the corresponding deviation (*rms*) is down to 2.11 × 10^−6^ and 3.35 × 10^−6^ for the dimension of 10^5^ and 10^6^, respectively. Due to our current computing conditions, we could not make more calculations for higher dimensional matrices, however since various matrices have been tested in this work, we believe that, our conclusion is reliable even for larger dimensions.

It needs to be pointed out that, although diagonal elements are generated using the same way as off-diagonal ones, the above conclusions are irrelevant to the sign of diagonal elements. It can be proved as follows: suppose $\bar{H}$ being a random real symmetric matrix with non-positive off-diagonal elements but some positive diagonal elements, then $\bar{H}$ can always be expressed as *H*_*r*_ + *d* × *I*. Here *H*_*r*_ is a RRSM with both diagonal and off-diagonal elements being non-positive, *I* is an identity matrix, and *d* is a positive constant. It is self-evident that, $\bar{H}$ and *H*_*r*_ share the same eigenvectors, since *D* × *I* just simultaneously shifts all diagonal elements. Thus the linear relationship discussed above holds for $\bar{H}$ if *H*_*r*_ does.

The linear relationship presented in RRSMs should have both physics and mathematical origins. Unfortunately, neither simple mathematics theorem nor physical theory can provide definite information regarding to this issue. However, we still can make some arguments from both physical and mathematics considerations as follows. For RRSMs, according to the Perron-Frobenius theorem^42,43^, all *g*_*i*_ can be taken as real and have the same sign. For convention, we take *g*_*i*_ > 0. The ground state energy can be directly calculated by

2 $$E= < G| H| G > =({\sum }_{i}{g}_{i} < {e}_{i}| )H({\sum }_{j}{g}_{j}| {e}_{j} > )={\sum }_{i,j} < {e}_{i}| H| {e}_{j} > {g}_{i}{g}_{j}={\sum }_{i,j}{H}_{ij}{g}_{i}{g}_{j},$$

under the mean-field-like approximation, we can rewrite the energy of ground state as

3 $$\begin{array}{ll}E & =\,{\sum }_{i,j}{H}_{ij}{g}_{i}{g}_{j}\to {\sum }_{i,j}{H}_{ij}{g}_{i} < g > = < g > {\sum }_{i}\left({g}_{i}{\sum }_{j}{H}_{ij}\right)\\  & = < g > {\sum }_{i}{g}_{i}{S}_{i}=- < g > {\sum }_{i}{g}_{i}(-{S}_{i})=- < g > \overrightarrow{g}\cdot \overrightarrow{S},\end{array}$$

where < *g* > refers to the mean value, *i*. *e*. , $< g > =\frac{1}{N}{\sum }_{j}{g}_{j}$. In the above equation the energy *E* is expressed as the scalar product of two identity vectors, $\overrightarrow{g}$ and $\overrightarrow{S}$. Here $\overrightarrow{g}={[{g}_{1},{g}_{2},\cdots ,{g}_{N}]}^{{\rm{T}}}$ and $\overrightarrow{S}={[-{S}_{1},-{S}_{2},\cdots ,-{S}_{N}]}^{{\rm{T}}}$. Since the ground state has the minimum energy, $\overrightarrow{g}$ should be equal to $\overrightarrow{S}$, namely *g*_*i*_ = −*S*_*i*_.

As the further remark, we should discuss in which conditions the linear relationship will be broken down. Firstly, for diagonal dominated matrices, in which the distribution width of diagonal elements is larger than the sum of off-diagonal ones, the linear relationship breaks down, but the positive correlation keeps. Here the positive correlation means that *g*_*i*_ decreases with the increase of *S*_*i*_. Secondly, for some band matrices with non-positive off-diagonal elements, again the linear relationship breaks down, but the positive correlation holds. It needs to be addressed that, although the current results are obtained for RRSMs, our preliminary results show that, the positive correlation between *g*_*i*_ and *S*_*i*_ may still be kept if the sum of negative elements prevails over the sum of positive ones in a matrix. Surely the correlation may be complicated rather than simple linearity. For more general cases, the further investigations are worth doing^44^.

### Application on specific models

The linear relationship obtained in present work should have broad applications for many physics systems of practical interests, in which the Hamiltonian matrices are similar to RRSMs. The applications are manifold. It can be used to determine the energy and wave function of ground states, or to analyze the physical properties of ground states. This strategy has the advantages of briefness, high efficiency and simplicity to be generalized to arbitrary large dimensions. Of course, the most powerful application maybe combining the linear relationship with other modern matrix diagonalization techniques, such as the Monte Carlo method, to calculate the properties of ground state.

Although the conclusion is obtained based on random matrices, the linear relationship still keeps for non-random matrices, in which off-diagonal elements are non-positive. As practical examples, we have tested the linear relationship on both one-dimensional Hubbard model^45^ and quantum Ising model^46–48^. The Hubbard model is extremely fundamental and important for a variety of areas, especially in the study of strongly correlated quantum systems. It is an important model to describe metal-insulator transitions^49^ and to understand high temperature superconductors^50,51^. The quantum Ising model, or equivalently, the Ising model in a transverse field, is one of the most widely used paradigm in studying quantum phase transitions^48,52,53^.

At present step, the aim is to examine the validity of the linear relationship for quantum many body models, therefore the calculations are restricted to relatively small systems. For the one-dimensional 4-site Hubbard model, the Hamiltonian matrix is a 36 × 36 one, which can be directly diagonalized. If anti-periodic boundary conditions are used, all off-diagonal elements are non-positive. The on-site coupling strength is chosen to be *U*/*t* = 0 and *U*/*t* = 1 respectively. For the larger *U* situations, it corresponds to diagonally dominant matrices, which is beyond the scope of current work. For the one-dimensional quantum Ising model^48^, the Hamiltonian reads ${H}_{I}=-g{\sum }_{i}{\widehat{\sigma }}_{i}^{x}-{\sum }_{ < ij > }{\widehat{\sigma }}_{i}^{z}{\widehat{\sigma }}_{j}^{z}$, where ${\widehat{\sigma }}_{i}^{x}$ and ${\widehat{\sigma }}_{i}^{z}$ are Pauli matrices and *g* > 0 is a dimensionless parameter. In the basis where ${\widehat{\sigma }}_{i}^{z}$ is diagonal, the off-diagonal elements of the Hamiltonian matrix of *H*_*I*_ are constituted by −*g* and 0. The large *g* corresponds to the case, in which the Hamiltonian matrix is not diagonally dominant and the linear relationship should be kept. In current studies, *g* = 10 and periodic boundary conditions are adopted. The system size (length of chain) considered includes *L* = 4, 6, 8, 10, 12, 14. In order to use the linear relationship, we assume *g*_*i*_ = *c*_1_*S*_*i*_ + *c*_2_ = *c*_1_(*S*_*i*_ + *c*_2_/*c*_1_), obviously only *c*_2_/*c*_1_ is important for physical wave functions, thus the energy of ground state can be obtained by means of variation respect to the parameter *c* = *c*_2_/*c*_1_.

**Figure 5:**
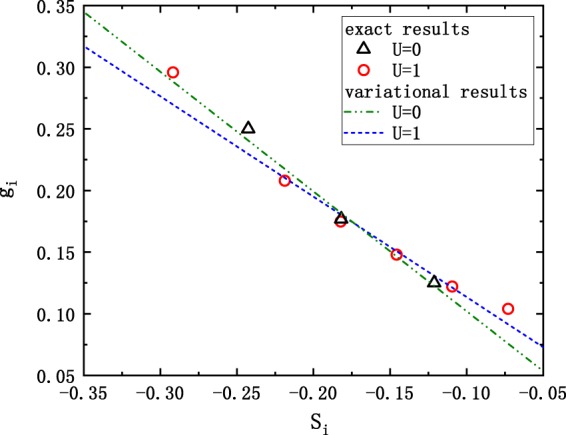
Element of the eigenvector for the minimum eigenvalue (*g*_*i*_) versus the sum of matrix elements in corresponding row (*S*_*i*_) for one-dimensional 4-site half-filled Hubbard model. The black △ and red ◦ correspond to the coupling strength of *U*/*t* = 0 and *U*/*t* = 1 respectively. The dash-dot-dot and dashed lines are variational results based on linear relationship for *U*/*t* = 0 and *U*/*t* = 1 respectively. Approximate linear relationships can be observed, which are in agreement with our predication.

Figure 5 presents the coefficients of wave-function of ground state for the Hubbard model, in which the dashed lines and symbols are the variational and exact values respectively. One can see that, although the corresponding matrices for both *U*/*t* = 0 and *U*/*t* = 1 cases are not random, the clear linear relationship between *g*_*i*_ and *S*_*i*_ holds, which is in agreement with our predication. And the ground-state energy obtained according to the linear relationship is much close to the exact value as shown in Table 1.

**Table 1:** The results of variational parameter (*c*) and calculated ground state energy for both the one-dimensional Hubbard and the quantum Ising model. The ground state energies obtained according to the linear relationship (*E*_scaling_) are much close to the exact value (*E*_exact_). Here the exact energy is obtained by direct diagonalization.

	Quantum Ising Model						Hubbard Model	
	*L* = 4	*L* = 6	*L* = 8	*L* = 10	*L* = 12	*L* = 14	*U* = 0	*U* = 1
*c*	0.000620	−0.0413	−0.0311	−0.0187	−0.0104	−0.00556	0.00954	−0.0137
*E*_scaling_	−10.024938	−10.024907	−10.024876	−10.024845	−10.024815	−10.024785	−1.41202	−1.17314
*E*_exact_	−10.024938	−10.025015	−10.025016	−10.025016	−10.025016	−10.025016	−1.41421	−1.18082

**Figure 6:**
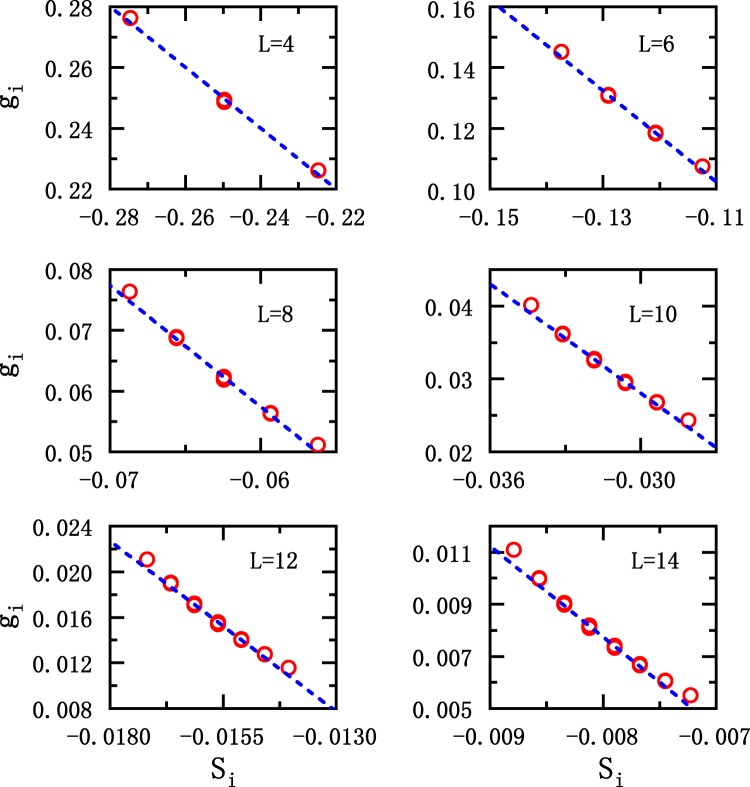
Similar to Fig. 4, but the results are corresponding to the one-dimensional quantum Ising model for system sizes of *L* = 4, 6, 8, 10, 12, 14 respectively. The dashed lines are variational results based on linear relationship, and the symbols represent the exact results.

Similar to Fig. 5, Fig. 6 presents the results for the one-dimensional quantum Ising model. Clearly, the linear correlation between *g*_*i*_ and *S*_*i*_ is still pronounced. It can be seen that, the ground state energy obtained according to the linear relationship is quite accurate as listed in Table 1, the errorbar is less than 0.01%.

## Discussion and Summary

Besides of direct approximations to ground state eigenvectors, the scaling relationship proposed in this article may also be used to improve the performance of other numerical methods. It is well known that, the appropriate choice of an initial wave function is one of the most common challenges in many computational methods, such as quantum Monte Carlo, density matrix renormalization group, as well as the power method. As a by-product, at least for one kind of matrices discussed currently, our scaling relationship points out how to obtain an optimal initial wave function.

In summary, we have explored the probability to establish an immediate connection between the eigenvector and matrix elements. At least, for real symmetric random matrices with non-positive off-diagonal elements, this kind of connection has been figured out. Namely, the eigenvector of ground states can be directly obtained by the sum of matrix elements in each row. This connection provides a feasible method to calculate the eigenvector of matrices without diagonalization. Although the linear relationship is obtained based on the random matrices, the test on model systems further confirms the validity of the scaling between the ground state eigenvector and matrix elements.

## Methods

The dimension of RRSMs considered in this work ranges from 100 to 10^6^. Around ten thousand matrices are calculated for each dimension. All matrix elements are generated according to two types of distribution, *i*. *e*, the uniform and Gaussian distribution. For the uniform distribution, a number in the range of [*X*_*min*_, 0], where *X*_*min*_ is a negative number, is randomly chosen and assigned to a matrix element. For the Gaussian distribution, a matrix element is generated through the Gaussian distributions, in which the Gaussian variation and mean value are set as 1.0 and −2.0 respectively. In Gaussian distribution, a few percent of off-diagonal elements are positive, however this situation does not alter our conclusions. To further check the universality of our results, the matrix elements in several rows are also randomly enlarged or reduced. And the effect of the matrix density (*ρ*) (e.g., the number of non-zero elements divided by the total number of elements) is also investigated. To reduce the matrix density, a certain amount of randomly chosen matrix elements are taken to be zero.

For each matrix, the ground state is obtained by direct diagonalization firstly, then the normalized *g*_*i*_ and *S*_*i*_ are calculated straightforwardly. The values of *g*_*i*_ and *S*_*i*_ are normalized. In quantum physics, the square of *g*_*i*_ corresponds to the probability of finding the *i*-th sate, therefore the normalization of *g*_*i*_ is necessary. Additionally, without the normalization, *S*_*i*_ may vary significantly for different matrices. In this case, although the scaling relationship between *g*_*i*_ and *S*_*i*_ still holds, the relationship will vary for different matrices. However, if both *g*_*i*_ and *S*_*i*_ are re-scaled according to the normalization condition, the scaling relationship will be universal, which is the key results of current work.

## Data Availability

All relevant data are within the paper.
